# The relationship between dietary intake of B vitamins and H. pylori seropositivity

**DOI:** 10.1371/journal.pone.0336471

**Published:** 2025-12-17

**Authors:** Yuexia Lu, Ting Wang, Shuaipeng Yuan, Jiqi Ouyang, Jinsheng Dong, Xin Jiang, Xiao Shao, Huazhao Xu, Runshun Zhang

**Affiliations:** 1 Guang ‘anmen Hospital, China Academy of Chinese Medical Sciences, Beijing, China; 2 Institute of Basic Research in Clinical Medicine, China Academy of Chinese Medical Sciences, Beijing, China; King Fahad Medical City, SAUDI ARABIA

## Abstract

**Introduction:**

The association of vitamins with *Helicobacter pylori (H. pylori)* infection is of great interest yet currently remains controversial. Most studies have focused on serum levels of vitamins, whereas we aimed to detect a correlation between dietary intake of B vitamins and *H. pylori* infection.

**Methods:**

We accessed the National Health and Nutrition Examination Survey (NHANES) of U.S. citizens and employed the Full Sample 2-Year mobile examination center (MEC) Examination Weight for data analysis For the 1999–2000 NHANES data period, 3,485 U.S. citizens made up our study.This cross-sectional study analyzed the relationship between dietary B vitamins and *H. pylori* seropositivity through weighted logistic regression. Variables were used to perform subgroup analyses, and the presence of linear correlation was explored using restricted cubic spline (RCS) plot progression.

**Results:**

Participants with *H. pylori* infections displayed lower intakes of vitamins B1, B2, niacin, B6, folate, and B12 compared with those without *H. pylori* infection. The Odds Ratio values (95% Confidence Interval) of the highest quartile of dietary intake were 0.69 (0.48–0.98), 0.63 (0.49–0.81), 0.69 (0.48–0.97), 0.71 (0.52–0.98), 0.67 (0.51–0.88), and 0.93 (0.66–1.29) for Log Vitamin B1, Log Vitamin B2, Log Niacin, Log Vitamin B6, Log Folate, and Log Vitamin B12, respectively, in comparison to the lowest quartile. Age and ethnicity were found to influence the association between B vitamins consumption and *H. pylori* infection.

**Conclusion:**

The dietary intake of vitamins B1, B2, niacin, B6, and folate were inversely correlated with the probability of an *H. pylori* infection. The mechanisms underlying this association may involve gastrointestinal flora, oxidative stress, as well as immune and mitochondrial regulation.

## 1. Introduction

In 1984, Marshall and Warren discovered *Helicobacter pylori (H. pylori)*, a gram-negative microaerobic bacterial species that colonized the stomach mucosa [1]. *H. pylori* infection is closely related to the incidence of peptic ulcer, acute and chronic gastritis, mucosa-associated lymphoid tissue lymphoma and gastric cancer. Further, it may increase the risk of esophageal adenocarcinoma and exacerbate the progression of severe pancreatitis [2–4]. The World Health Organization has classified it as a class I carcinogen [5]. At present, approximately 4.4 billion people worldwide(half of the world population) are infected with *H. pylori*, which has brought a heavy public health burden [6]. Although antibiotic treatment is the mainstay of *H. pylori* infection management, dietary prophylaxis and treatment have attracted much attention due to the increase in antibiotic resistance.

B vitamins, obtained from food, are water-soluble and widely found in yeast, vegetables, and grains. These vitamins are enzymatic cofactors in human metabolic reactions, which are involved in energy metabolism, production of neurotransmitters, DNA synthesis and repair, and can reduce oxidative stress [7–10] and enhance human immunity [11,12]. Oxidative stress can promote the onset and development of *H. pylori* infection [13], while an adequate intake of dietary antioxidants may help reduce this risk [14]. B vitamins may counter *H. pylori* colonization of the gastric mucosa by increasing patient immunity and decreasing oxidative stress, and some studies have found that niacin intake in patients with *H. pylori* infection was lower than that in non-infected patients [15]. However, current research on the association between B vitamins and *H. pylori* infection has focused on vitamin B12, niacin, and folate, although this correlation remains controversial. Additionally, most studies measure serum vitamin levels and lack the inclusion of large-scale data on the association between dietary intake of B vitamins and *H. pylori* infection.

Therefore, we employed large-scale sample data to investigate an association between the dietary intake of B vitamins in the diet and the occurrence of *H. pylori* infection. It is hoped that our present study may provide some thoughts and suggestions on dietary management for the prevention and treatment of H. pylori infections.

## 2. Materials and methods

### 2.1. Data source and study population

The National Health and Nutrition Examination Survey (NHANES) is an invaluable resource for the investigation of the health status and nutritional status of the U.S. It uses a stratified, multi-stage sampling design, which is representative of the U.S. population. Approval for the database from the Ethics Review Board at the National Centre for Health Statistics (NCHS) was obtained. All participants provided written informed consent. We used the Full Sample 2-Year mobile examination center (MEC) Examination Weight for data analysis..

For the 1999–2000 NHANES data period, 9,965 individuals participated, and after removing those who were missing anthropometric information, questionnaire responses, or blood biochemical indicators, 3,485 US citizens made up our research sample.

### 2.2. *H. pylori* seropositivity

The *H. pylori* immunoglobulin G (IgG) enzyme-linked immunosorbent assay (ELISA) developed by Wampole Laboratories subjectively identifies and quantifies *H. pylori-*targeted IgG antibodies in human serum [16]. Using established ELISA standards, study participants were categorized as either serologically positive (optical density (OD > 1.1) or serologically negative (OD < 0.9) for *H. pylori*. To prevent statistical results from being deceptive, fuzzy values (0.9–1.1) were not included in the analysis [17].

### 2.3. Dietary intake of B vitamins

The MEC staff conducted in-person interviews to gather information on dietary nutrient intake [18]. Details about every food and drink the survey participant consumed has had in the last 24 hours were recorded via the NHANES Dietary Recall Method. This enabled the acquisition of thorough dietary data, which NHANES personnel evaluated and validated subsequently.

To mitigate recall bias and other effects of the 24-hour dietary recall method, NHANES adopted the following measures: First, after the dietary recall procedure, respondents were requested to complete a brief “post-recall” questionnaire. This dietary recall questionnaire was conducted after the 24-hour recall procedure. Second, a dual recall approach was employed, with at least 10% of the representative sample randomly selected for a second independent dietary recall interview. The second interview could be conducted either face-to-face or by telephone [19,20].

In this study, we focused on the dietary intake of B vitamins intake from food sources. The quartiles (Q1-Q4) indicate different levels of B vitamins intake, with thresholds of 25%, 50%, and 75% of the total sample intake, respectively.

### 2.4. Covariates

The study incorporated a range of covariates, including race, age, gender, education, poverty-to-income ratio, household size, body mass index, triglycerides, total cholesterol, C-reactive protein, smoking, alcohol consumption, hypertension, diabetes mellitus, cardiovascular disease, and stroke disease [21–23].Participants were classified by race as Mexican American, non-Hispanic white, non-Hispanic black, or other. Education was categorized as below high school, high school, and above high school. Household size was classified as Small (1–3 members) and Large (4 or more members). Hypertension, diabetes mellitus, and stroke disease were obtained from a questionnaire that asked participants whether they had ever been diagnosed by a doctor with these diseases. Cardiovascular disease was determined by questionnaires about congestive heart failure, coronary heart disease, angina/angina, or heart attack. Smoking status and alcohol consumption were categorized into three sections: never, former and current [24]. Triglycerides, total cholesterol, and C-reactive protein levels were obtained from blood samples taken according to standard MEC procedures. Age, poverty-to-income ratio, and body mass index were continuous variables.

### 2.5. Statistical analysis

Categorical variables were expressed as percentages (%), while continuous variables were summarized using the mean (standard error SE) or median (interquartile range IQR). Normally distributed data were analyzed using the t-test, the Wilcoxon rank-sum test for skewed data, and the Chi-square test for categorical variables.

Given the skewed distribution of B vitamins intake levels, we transformed them into natural logarithms to normalize the data. The transformed data allowed the application of parametric statistics assuming normality. Subsequently, the logarithm of the B vitamins intake level was divided into quartiles for further analysis. Before undertaking multivariate analyses, we performed univariate weighted logistic regression analyses to assess the associations of each variable separately with *H. pylori* infection status. We used weighted logistic regression to analyze the association between B vitamins intake and *H. pylori* infection. Subgroup analyses stratified all confounders, and p-values for interactions were used to determine whether stratification effects were significant.Restricted cubic spline (RCS) regression analysis examined whether there was a linear relationship between the dietary intake levels of B vitamins and *H. pylori* seropositivity, adjusting for confounding variables.A multiple comparison correction method was used to observe whether the corrected quartile model was significant.

## 3. Result

### 3.1. Basic demographic characteristics

**Fig 1:**
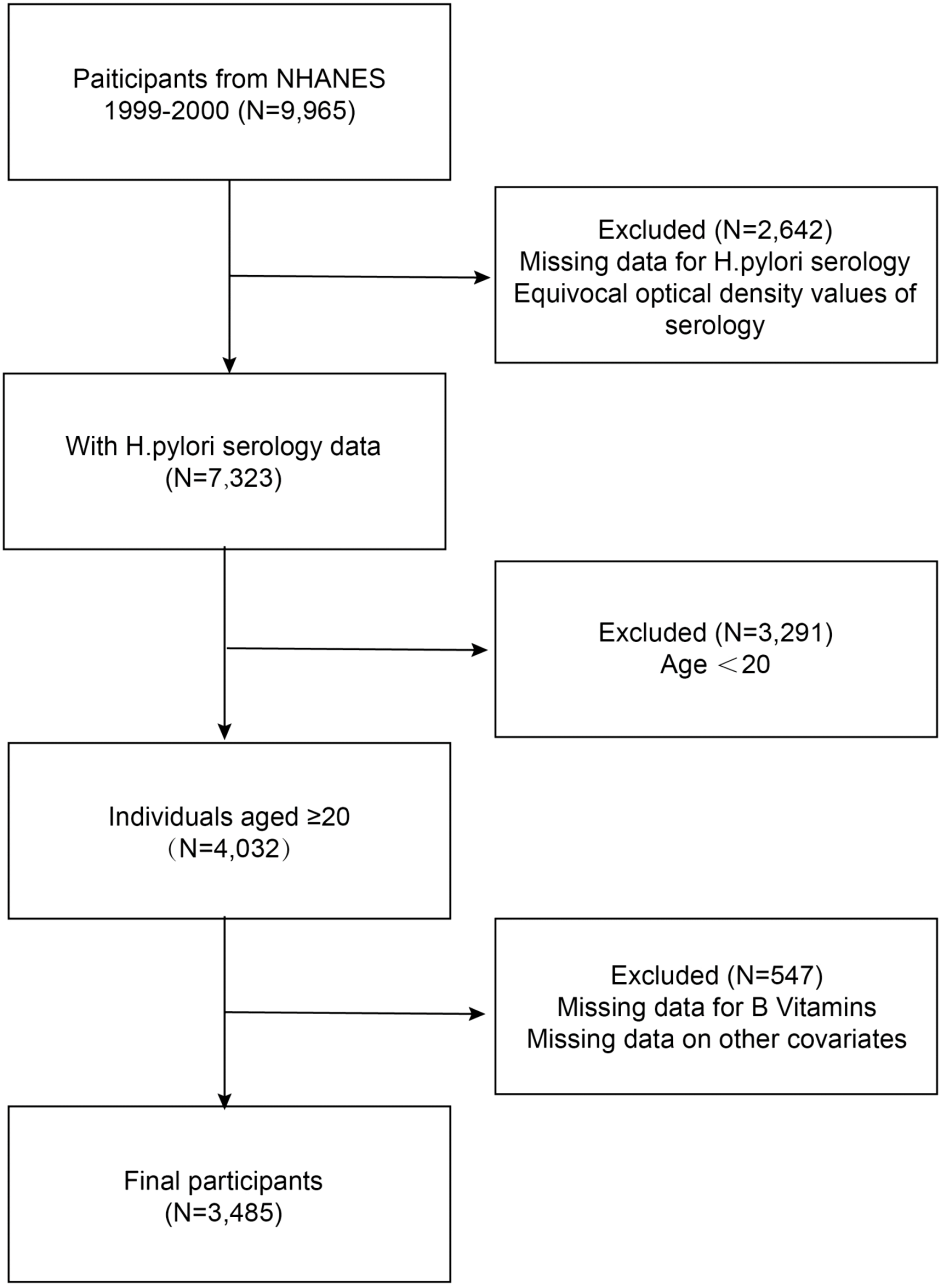
The process of selecting the study population.

This study involved 3,485 adults, aged between 20 and 85 years. **Fig 1** illustrates the inclusion and exclusion criteria for this study.

**Table 1** displays the participants according to either *H. pylori* infection.In total, 1,508 participants were identified as being infected with *H. pylori*. The prevalence rate for *H. pylori* infection was 43.27%. The mean age of participants with *H. pylori* seropositivity was 48.79 years, which was older than those who were negative for *H. pylori* infection. In contrast, individuals seropositive for *H. pylori* had a reduced percentage of non-Hispanic whites and significantly lower intakes of B vitamins (all p < 0.05). A natural logarithmic transformation was performed on the B vitamins intake values in order to approximate a normal distribution for analysis. Even when log- transformed,participants seropositive for *H. pylori* had significantly lower intakes of B vitamins.

**Table 1:** Baseline characteristics of the study population (N = 3,485).

Variable	Total (n = 3485)	Helicobacter pylori Seronegativity (n = 1,977)	Helicobacter pylori Seropositivity (n = 1,508)	p value
**Gender, n(%)**				0.922
Male	1,704 (50.05)	939 (50.01)	765 (50.15)	
Female	1,781 (49.95)	1,038 (49.99)	743 (49.85)	
**Race, n(%)**				<0.001
Mexican American	918 (5.85)	308 (3.23)	610 (12.00)	
Other	331 (12.52)	146 (8.43)	185 (22.12)	
Non-Hispanic white	1,643 (72.27)	1,257 (81.82)	386 (49.87)	
Non-Hispanic black	593 (9.36)	266 (6.52)	327 (16.01)	
**Education, n(%)**				<0.001
Below high school	1,264 (22.36)	437 (14.97)	827 (39.69)	
High school	792 (25.86)	502 (25.78)	290 (26.03)	
Above high school	1,429 (51.79)	1,038 (59.25)	391 (34.28)	
**Age, Mean (SE)**	44.94 (0.43)	43.29 (0.53)	48.79 (0.62)	<0.001
**Smoke, n(%)**				0.004
Never	1,827 (50.63)	1,072 (52.92)	755 (45.27)	
Former	956 (25.35)	535 (25.23)	421 (25.65)	
Current	702 (24.01)	370 (21.85)	332 (29.08)	
**Stroke, n(%)**	112 (2.06)	52 (1.81)	60 (2.67)	0.057
**CVD, n(%)**	298 (6.49)	146 (5.68)	152 (8.41)	0.012
**DM, n(%)**	313 (5.63)	137 (4.67)	176 (7.87)	0.016
**HBP, n(%)**	1,051 (23.82)	542 (21.99)	509 (28.11)	0.007
**Household size, n(%)**				0.028
Small (1–3members)	2,249 (67.28)	1,351 (68.81)	898 (63.71)	
Large (4 or more members)	1,236 (32.72)	626 (31.19)	610 (36.29)	
**Drink, n(%)**				<0.001
Never	547 (12.49)	289 (11.80)	258 (14.12)	
Former	596 (13.09)	284 (11.02)	312 (17.94)	
Current	2,342 (74.41)	1,404 (77.17)	938 (67.94)	
**PIR, Mean (SE)**	2.90 (0.11)	3.10 (0.11)	2.41 (0.10)	<0.001
**TG, Mean (SE)**	139.47 (3.10)	136.63 (3.14)	146.13 (4.84)	0.062
**TC, Mean (SE)**	203.46 (1.16)	203.28 (1.56)	203.88 (2.26)	0.844
**CRP, Mean (SE)**	0.41 (0.01)	0.40 (0.01)	0.44 (0.02)	0.091
**BMI, Mean (SE)**	27.86 (0.21)	27.83 (0.27)	27.94 (0.19)	0.712
**Vitamin B1, M (Q₁, Q₃)**	1.47 (1.01, 2.11)	1.53 (1.05, 2.18)	1.37 (0.91, 1.92)	<0.001
**Vitamin B2, M (Q₁, Q₃)**	1.76 (1.22,.52)	1.84 (1.29, 2.62)	1.53 (1.06, 2.20)	<0.001
**Niacin, M (Q₁, Q₃)**	21.48 (14.52, 30.17)	22.66 (15.21, 31.39)	19.20 (13.36, 26.94)	<0.001
**Vitamin B6, M (Q₁, Q₃)**	1.65 (1.09, 2.40)	1.74 (1.13, 2.46)	1.52 (1.00, 2.23)	<0.001
**Folate, M (Q₁, Q₃)**	333.19(226.42, 482.60)	347.40 (240.52, 502.53)	294.67(189.25, 446.81)	<0.001
**Vitamin B12, M (Q₁, Q₃)**	3.57 (2.00, 5.76)	3.73 (2.04, 5.93)	3.16 (1.89, 5.28)	0.005
**Log Vitamin B1, Mean (SE)**	0.35 (0.03)	0.39 (0.03)	0.25 (0.03)	<0.001
**Log Vitamin B2, Mean (SE)**	0.53 (0.02)	0.59 (0.03)	0.38 (0.02)	<0.001
**Log Niacin, Mean (SE)**	3.02 (0.02)	3.07 (0.02)	2.89 (0.03)	<0.001
**Log Vitamin B6, Mean (SE)**	0.45 (0.02)	0.50 (0.03)	0.35 (0.04)	0.002
**Log Folate, Mean (SE)**	5.76 (0.03)	5.81 (0.04)	5.63 (0.04)	<0.001
**Log Vitamin B12, Mean (SE)**	1.16 (0.03)	1.22 (0.04)	1.04 (0.04)	0.008

CVD: cardiovascular disease; DM: diabetes mellitus; HBP: High Blood Pressure; PIR: the ratio of family income to poverty; TG: triglyceride; TC: Total cholesterol; BMI, body mass index; CRP: C-reactive protein.

### 3.2. Association between dietary intake levels of B vitamins and *H. pylori* infection

**Fig 2:**
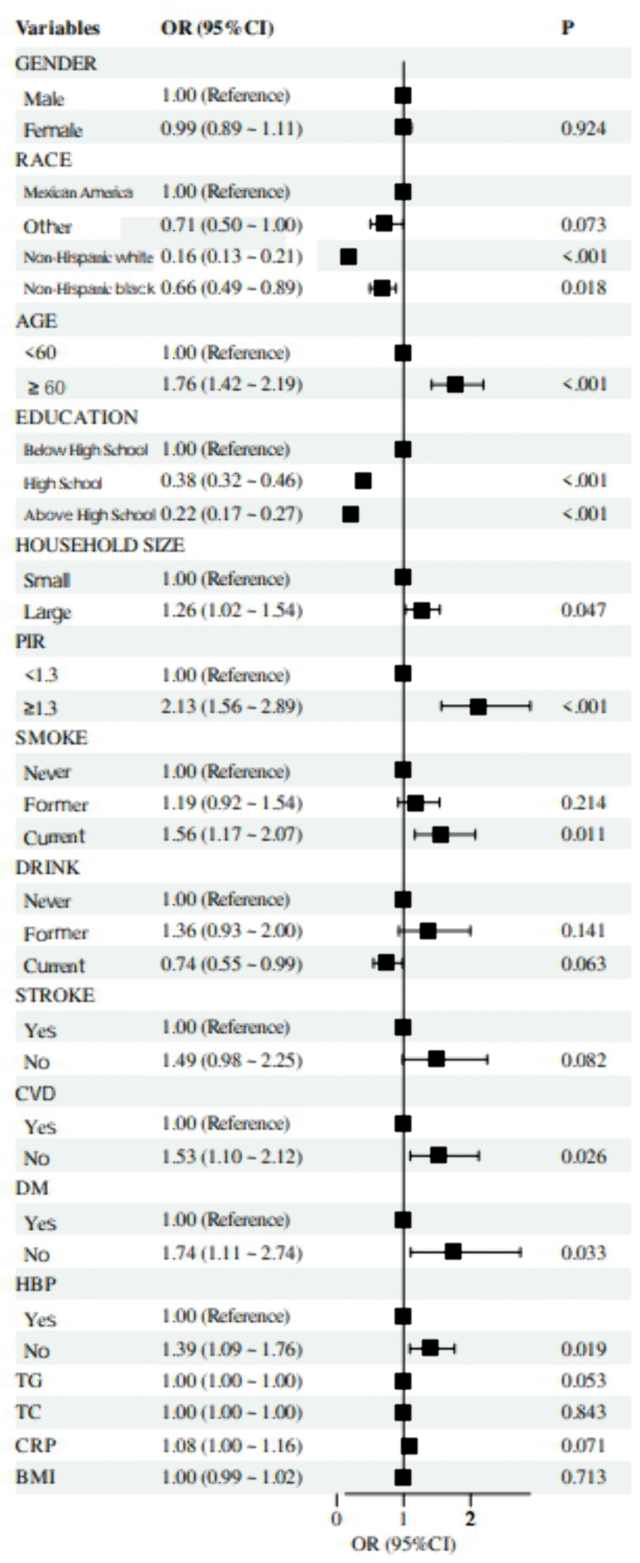
Forest plot displaying the associations between each covariate and H.pylori infection.

Firstly, to assess the relationship of each covariate with *H. pylori* seropositivity, univariate logistic regression analyses were performed (**Fig 2**). Individuals with a high school education or above were less likely to be infected with *H. pylori*, with an Odds Ratio (OR) of 0.38 and 0.22, respectively (all p < 0.001). Non-Hispanic whites had a significantly lower H. pylori prevalence compared with Mexican Americans (OR = 0.16, 95% Confidence Interval [CI]: 0.13–0.21, p < 0.001).

We found that the elderly (age ≥ 60 years) were more susceptible to an *H. pylori* infection. In addition, large-scale families, smokers, and people with cardiovascular, diabetic, and hypertensive comorbidities should be more concerned about an *H. pylori* infection.

(**Table 2**) shows the results of our multivariate logistic regression analyses. When B vitamins intake values were used as continuous variables in the log form, Model I (adjusted for no covariates) showed that a higher intake of B vitamins was associated with a lower probability of an *H. pylori* infection. However, the negative correlation between vitamin B12 intake and the prevalence of *H. pylori* infections was no longer statistically significant in Model II (adjusted for demographic factors) and Model III (adjusted for all covariates), and the quartile logistic regression findings were the same. As seen in Model III, each unit increase in the log intake value for vitamins B1, B2, niacin, B6, and folate, resulted in a reduction in the odds of *H. pylori* infection by 17%, 26%, 24%, 19%, and 26%, respectively, (ORs 0.83, 0.74, 0.76, 0.81 and 0.74, respectively).

**Table 2:** Weighted logistic regression of the association between log Vitamins intake and H. pylori Infection.

	Model I		Model II		Model III	
	OR (95% CI)	p value	OR (95% CI)	p value	OR (95% CI)	p value
**Log Vitamin B1(mg/d)**	0.71(0.56,0.85)	0.001	0.75(0.62,0.91)	0.007	0.83(0.70,0.99)	0.044
**Log Vitamin B2(mg/d)**	0.57(0.48,0.69)	<0.001	0.67(0.56,0.80)	<0.001	0.74(0.62,0.88)	0.003
**Log Niacin(mg/d)**	0.62(0.52,0.75)	<0.001	0.68(0.56,0.84)	0.002	0.76(0.62,0.93)	0.012
**Log Vitamin B6(mg/d)**	0.71(0.59,0.85)	0.001	0.72(0.59,0.88)	0.003	0.81(0.68,0.96)	0.017
**Log Folate(ug/d)**	0.65(0.55,0.77)	<0.001	0.66(0.56,0.79)	<0.001	0.74(0.61,0.89)	0.004
**Log Vitamin B12(ug/d)**	0.85(0.75,0.95)	0.007	0.90(0.79,1.03)	0.107	0.91(0.80,1.05)	0.172
**Log Vitamin B1 quartiles**						
Q1(<−0.051)	1.0 [Ref]	1.0 [Ref]	1.0 [Ref]			
Q2(−0.051,0.344)	0.74(0.51,1.06)	0.094	0.80(0.53,1.19)	0.241	0.87(0.60,1.26)	0.425
Q3(0.344,0.693)	0.74(0.58,0.94)	0.018	0.78(0.58,1.05)	0.093	0.96(0.75,1.21)	0.681
Q4(>0.693)	0.53(0.39,0.74)	0.001	0.59(0.41,0.84)	0.007	0.69(0.48,0.98)	0.037
**Log Vitamin B2 quartiles**						
Q1(<0.14)	1.0 [Ref]	1.0 [Ref]	1.0 [Ref]			
Q2(0.14,0.501)	0.71(0.57,0.90)	0.006	0.76(0.56,1.03)	0.071	0.86(0.64,1.14)	0.266
Q3(0.501,0.859)	0.49(0.40,0.59)	<0.001	0.58(0.45,0.75)	<0.001	0.65(0.52,0.82)	0.001
Q4(>0.859)	0.42(0.32,0.54)	<0.001	0.54(0.42,0.69)	<0.001	0.63(0.49,0.81)	0.002
**Log Niacin quartiles**						
Q1(<2.606)	1.0 [Ref]	1.0 [Ref]	1.0 [Ref]			
Q2(2.606,2.976)	0.81(0.66,0.99)	0.045	0.86(0.68,1.08)	0.177	0.88(0.69,1.12)	0.272
Q3(2.976,3.327)	0.64(0.50,0.84)	0.003	0.72(0.52,0.98)	0.038	0.80(0.59,1.08)	0.127
Q4(>3.327)	0.49(0.37,0.65)	<0.001	0.59(0.43,0.81)	0.003	0.69(0.48,0.97)	0.035
**Log Vitamin B6 quartiles**						
Q1(<0.058)	1.0 [Ref]	1.0 [Ref]	1.0 [Ref]			
Q2(0.058,0.464)	0.82(0.58,1.16)	0.242	0.79(0.52,1.20)	0.245	0.88(0.62,1.24)	0.442
Q3(0.464,0.859)	0.72(0.56,0.92)	0.014	0.71(0.52,0.95)	0.027	0.83(0.64,1.07)	0.142
Q4(>0.859)	0.58(0.42,0.79)	0.002	0.60(0.42,0.86)	0.008	0.71(0.52,0.98)	0.041
**Log Folate quartiles**						
Q1(<5.366)	1.0 [Ref]	1.0 [Ref]	1.0 [Ref]			
Q2(5.366,5.777)	0.67(0.49,0.92)	0.017	0.70(0.49,1.01)	0.054	0.79(0.56,1.13)	0.182
Q3(5.777,6.156)	0.56(0.40,0.77)	0.002	0.59(0.40,0.87)	0.011	0.72(0.49,1.07)	0.094
Q4(>6.156)	0.52(0.39,0.70)	<0.001	0.56(0.42,0.73)	<0.001	0.67(0.51,0.88)	0.008
**Log Vitamin B12 quartiles**						
Q1(<0.621)	1.0 [Ref]	1.0 [Ref]	1.0 [Ref]			
Q2(0.621,1.191)	1.19(0.91,1.56)	0.194	1.21(0.90,1.64)	0.196	1.17(0.86,1.59)	0.299
Q3(1.191,1.677)	0.81(0.68,0.97)	0.023	0.90(0.73,1.11)	0.299	0.87(0.69,1.10)	0.219
Q4(>1.677)	0.74(0.56,0.96)	0.028	0.89(0.64,1.24)	0.474	0.93(0.66,1.29)	0.628

OR, odds ratio; CI, confidence interval; Ref: reference.

Model I: adjusted for no covariates.

Model II: adjusted for age, gender, race.

Model III: adjusted for all covariates.

We then divided the log B vitamins intake value from a continuous form into quartile forms(**Table 2**). In Model III, the fourth quartile had a lower rate of *H. pylori* infection compared with the first quartile, except for the vitamin B12 intake category. The fourth quartile results for the dietary intake of vitamins B1, B2, niacin, folate, and B6 were OR = 0.69(95% CI: 0.48–0.98), OR = 0.63(95% CI: 0.49–0.81), OR = 0.69(95% CI: 0.48–0.97), OR = 0.71(95% CI: 0.52–0.98), and OR = 0.67(95% CI: 0.51–0.88).

### 3.3. Subgroup analysis

**Fig 3:**
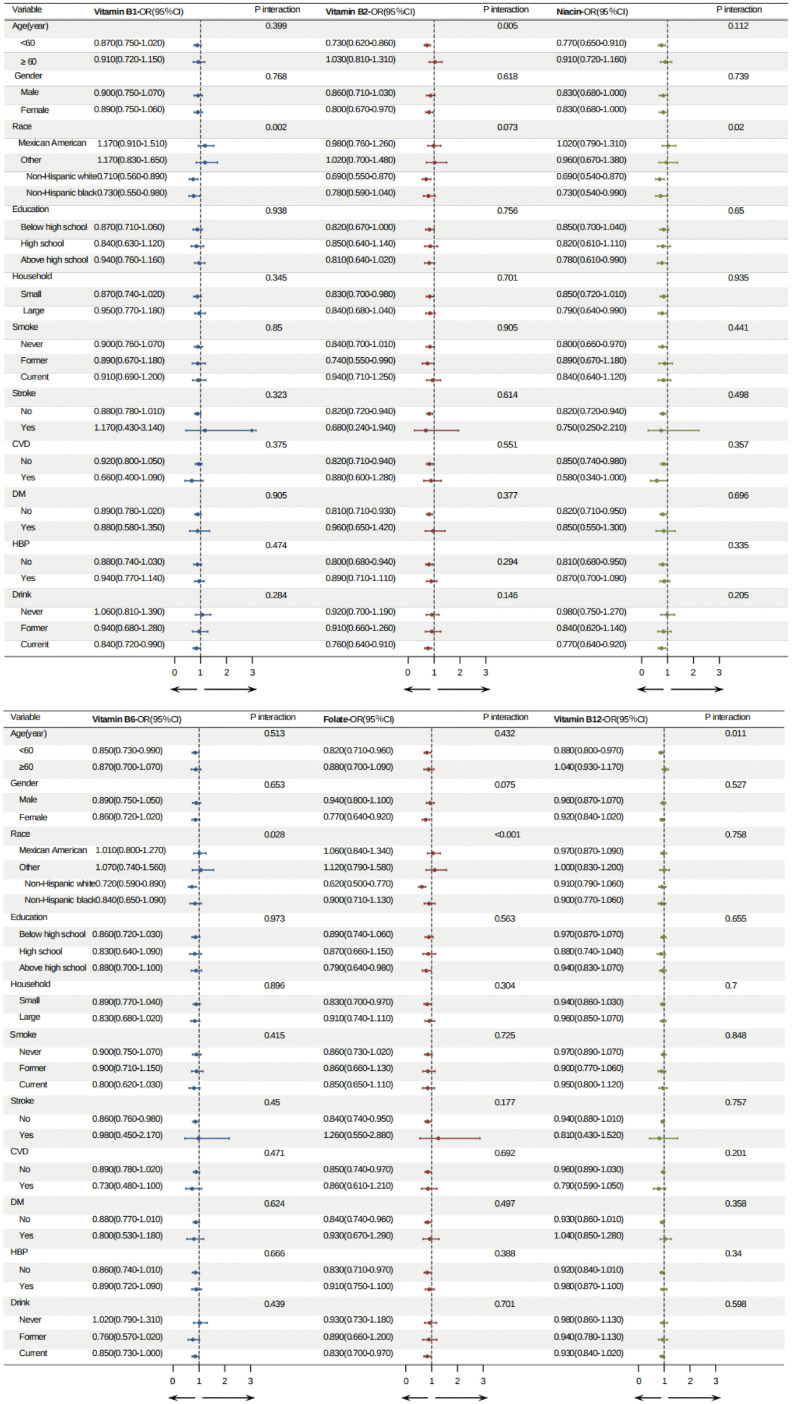
Forest plot demonstrated the relationship between Log B vitamins intake and H. pylori infection.

To assess the potential impact of the log B vitamins intake value on *H. pylori* infection, we performed stratified analyses for all covariates (**Fig 3**). No statistically significant interactions were found among the risk factor subgroups (all p-values for interactions >0.05). However, when stratified by demographics, the correlation between log vitamin B2 and B12 intake levels and *H. pylori* infection prevalence rates was found to be particularly influenced by age (its protective effect was significant in those under 60 years of age), and the correlation between log vitamin B1, niacin, B6, and folate intake levels and *H. pylori* infection was more strongly influenced by ethnicity. This suggests the need for tailored interventions for at-risk populations.

### 3.4. Dose-response relationships

**Fig 4:**
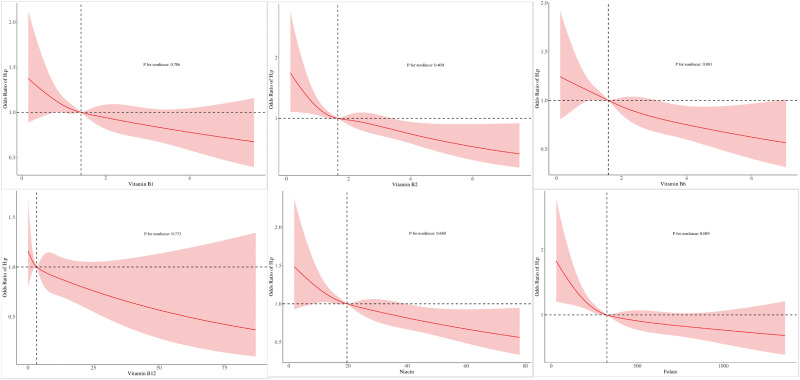
RCS demonstrated the linear/ nonlinear relationship.

To evaluate if there was a linear relationship between the dietary intake levels of B vitamins and the probability of an *H. pylori* infection, we used RCS analysis (**Fig 4**). The results showed a negative linear correlation between B vitamin intake and H. pylori seropositivity (p value >0.05).

### 3.5. Sensitivity analysis

To reduce the risk of Type I error (false positive), we performed a multiple comparison correction analysis on the quartile model (**Table 3**). False Discovery Rate (FDR) correction was prioritized to retain exploratory findings. After adjusting for covariates, the results of all B vitamins except vitamin B12 remained significant (q < 0.05), which is consistent with the research results in the article.

**Table 3:** FDR correction analysis on the quartile model.

Vitamin	FDR correction(q)
Vitamin B1	0.044
Vitamin B2	0.012
Niacin	0.042
Vitamin B6	0.049
Folate	0.024
Vitamin B12	0.628

## 4. Discussion

Our study findings confirmed that consuming vitamins B1, B2, Niacin, B6, and folate was inversely related to the probability of an *H. pylori* infection, with RCS plots showing a linear negative correlation. However, there was no evidence that vitamin B12 intake levels affected the prevalence of *H. pylori* infections after adjusting for confounders. Our subgroup analyses demonstrated that the association between B vitamins intake and *H. pylori* seropositivity was significantly influenced by ethnicity and age.The multiple comparison correction analysis shows that the results of all B vitamins except vitamin B12 remain significant.

Several previous studies have revealed a link between vitamins intake and *H. pylori* infection, Ebrahimi Z et al. [14] found that patients with *H. pylori* seropositivity had a lower intake of vitamin E and vitamin A and suggested that such antioxidants may help to prevent *H. pylori* attachment and colonization by enhancing the mucosal barrier. Chen Z et al. [15] suggested that increasing the dietary intake of niacin may reduce *H. pylori* infection rates. As for serum levels of vitamins, some studies have found that people with lower serum vitamin D levels were more likely to be infected with *H. pylori*, and vitamin D supplementation was recommended when treating *H. pylori* infection [25]. There has been a controversy regarding folate and vitamin B12: Some studies have found that serum folate and B12 levels were lower in patients with *H. pylori* infection compared with uninfected individuals [26], whereas others have not found an association between serum levels of B12, folate, and *H. pylori* infection [27,28]. A retrospective cohort study found significantly lower serum folate levels in patients with an *H. pylori* infection, and this relationship was not found for vitamin B12 [29]. Our present findings provide further evidence, especially in terms of dietary supplements:people who are not infected with *H. pylori* had a higher dietary intake of folic acid; however, no such association was found for dietary vitamin B12. Previous studies were mostly based on serum levels, while this study focused on dietary intake. Serum levels are affected by absorption and metabolism and may underestimate the role of diet. In addition, differences in the study samples’ populations may also lead to differences in research results.In future, large and rich race cohort studies are needed to explain the effect of vitamins on *H. pylori* infection.

Although the mechanisms underlying the relationship between B vitamins intake and an *H. pylori* infection remain unclear, our current findings are biologically plausible. First, Vacuolating cytotoxin A (VacA) and Cytotoxin-associated gene A are two important virulence factors of *H. pylori*, which can help *H. pylori* to successfully colonize and promote inflammatory response and cancer by disrupting the function of gastric epithelial cells [30].Vitamins B1, B2, niacin, and folate are the cofactors of various enzymes in the human body that aid in the production of sodium butyrate, which inhibits the production of H. pylori virulence factor and pro-inflammatory factor IL-8 [31]; at the same time, it increases beneficial biological flora and builds an environment unfavorable for *H. pylori* colonization [32–34]. In addition, pyridoxal phosphate, the active form of vitamin B6, competes with guanosine-5 ‘-monophosphate to form a Schiff base with a key amino acid residue (Lys322 residue) in the adenylosuccinate synthetase of *H. pylori* [35]. It has direct antibacterial action against *H. pylori*. Second, *H. pylori* induces the production of large quantities of reactive oxygen species (ROS) in the host, leading to oxidative stress and damage to normal cells, and resulting in disease progression [36]. B vitamins may inhibit *H. pylori* from surviving or proliferating by decreasing oxidative stress, which prevents this bacterium from adapting to the gastric environment. Although the major function of vitamin B1 and vitamin B2 is to participate in energy metabolism, they exert their antioxidant effects indirectly through these metabolic processes. Vitamin B1 can enhance the activity of antioxidant enzymes such as superoxide dismutase [37], and vitamin B2 can regenerate other antioxidants, like glutathione (GSH) and vitamin C [38]. Niacin and vitamin B6 act as antioxidants, protecting cells from oxidative damage through the reduction of ROS [39–41]. Folate is involved in one-carbon metabolism, supports GSH synthesis, and reduces oxidative stress as well as oxidative stress-induced cellular damage by maintaining mitochondrial health and reducing ROS production [42,43]. Third, *H. pylori* colonization of the gastric mucosa reduces the host immune response and promotes immune escape through multiple pathways [44,45]. B vitamins are essential for the maturation and functioning of T and B cells, which enhance human immune function and reduce the risk of infection [46]. Finally, control of mitochondrial function has been reported to be an efficacious therapeutic target to combat bacterial infections [47]. VacA reduces mitochondrial transmembrane potential and induces mitochondrial dysfunction and energy deficiency [48]. B vitamins may correct VacA toxicity in gastric mucosal cells by regulating the signal transduction of mitochondrial metabolism to the nucleus, and inhibiting the colonization effect of *H. pylori* on gastric epithelial cells [49].More animal or cell culture studies are needed in the future to verify these mechanisms.

Of note, we found the lowest *H. pylori* seroprevalence among non-Hispanic whites, consistent with previous studies [50,51]. The correlation between the prevalence of an *H. pylori* infection and the dietary intake of vitamin B1, niacin, B6, and folate intake was significantly influenced by ethnicity. The negative correlation of the findings was stronger among non-Hispanic whites.The current research on the racial differences in *H. pylori* infection is not fully clear, but it is believed that the main causes are social economic and environmental factors, which are specifically reflected in the uneven distribution of medical resources and differences in living environments, etc. [52,53]. Studies suggest that these social economic reasons lead to disproportionately high incidence and mortality rates among minority races and ethnic groups in the United States [52]. Furthermore, the differences in gut microbiota may have led to the heterogeneity of the subgroups’ results. A study found that the structure of gut microbiota is significantly associated with race and ethnicity. Compared with Hispanic/Latino individuals, non-Hispanic white individuals have higher microbial diversity in their samples [54]. This diversity may be closely related to healthier metabolic functions, such as vitamin metabolism.The influence of the dietary intake of vitamin B2 and B12 on the prevalence of an *H. pylori* infection was only statistically significant in the non-elderly population. The incidence of vitamin B12 deficiency was found to be interrelated with type 2 diabetes mellitus as well as metformin use [55,56]. Therefore, the high prevalence of chronic diseases, the complexity of medication used by the elderly population and the decreased absorption of vitamins may have weakened the effect.Therefore, B vitamins need to be consumed as early as possible. Research on population differences in the effects of vitamins intake is limited, and more extensive studies are needed to tailor management programs to different populations. Our study has several highlights. First, this is the first study to assess an association between the dietary intake of B vitamins and the occurrence of an *H. pylori* infection in U.S. adults. Second, we used a large-scale, national data sample of the U.S. population, thereby increasing the reliability of our results. Third, we adjusted for several important confounders.

However, this study has some limitations. First, we were unable to exclude the effects of all confounding factors, although our study incorporated demographic characteristics, health behaviors and associated diseases.For example, key confounding factors such as occupation, dietary patterns, and other variables that may affect both B vitamins intake and the risk of an *H. pylori* infection may have introduced bias. Secondly,due to the limitations of cross-sectional studies, we were unable to determine causality. It is unclear whether an *H. pylori* infection is due to the low dietary intake of B vitamins or whether the reduced intake is influenced by factors such as the poor appetite or malabsorption in patients with an *H. pylori* infection.. Further studies such as cohort studies, intervention studies or Mendelian studies are needed in the future to clarify the causal relationship. Third, the NHANES lacks data on the intake of other B vitamins, such as vitamins B5 and B7. Finally, our dietary data were collected from 24-hour recall interviews, so there may be an error compared to the real intake.This may have affected the conclusions of the study. For example, the intake of B vitamins may have been overestimated, leading to conclusions that underestimated the role of B vitamins in reducing the probability of *H. pylori* infections.

Our research focused on the association between the dietary intake levels of B vitamins and *H. pylori* seropositivity. Although many researchers have found a negative correlation between vitamins and the probability of *H. pylori* infection, there were not many studies focusing on the original intake of vitamins, especially the comprehensive B vitamins. The findings of this study suggest that choosing foods rich in B vitamins may be beneficial for reducing the probability of *H. pylori* infection. Especially for people who have been infected multiple times, they can try to benefit from dietary choices and the cultivation of dietary habits.

## 5. Conclusion

This study provides supportive evidence for the notion that the dietary intake of vitamins may reduce the probability of *H. pylori* infection. We suggest that increasing the dietary intake of vitamins B1, B2, niacin, B6, and folate may reduce the probability of an *H. pylori* infection.Future Mendelian studies or prospective research are needed to verify the causal relationship and identify the optimal intake effect. Given the heterogeneity among subgroups based on the subgroup analysis results, further studies are needed to identify population-specific differences that regulate the impact of a dietary intake of B vitamins on *H. pylori* infections to optimize healthcare resource allocation and improve the effectiveness of *H. pylori* control.

## Supporting information

S1 Data Analysed data. (XLS)
